# It’s time to get serious about funding neighbourhood health care

**DOI:** 10.3399/BJGP.2025.0815

**Published:** 2026-03-01

**Authors:** Matthew Harris, Cornelia Junghans Minton, Caroline Taylor

**Affiliations:** 1 Clinical Reader in Public Health Innovation and Honorary Consultant in Public Health, Imperial College London, London, UK; 2 Senior Clinical Fellow and GP, Imperial College London and Milbank GP Practice, London, UK; 3 GP and former Clinical Chair of the National Association of Primary Care, London, UK

The NHS 10-year health plan, and its associated National Neighbourhood Health Implementation Programme (NNHIP), calls for a radical reorientation from hospital-based, reactive, and transactional services to a more relational, proactive, community-based, integrated support for people closer to, and indeed within, the home.^1^ Forty-three sites have already been selected as vanguards to spearhead the rollout of integrated neighbourhood health.^2^ Each will receive coaching support to bring about the much-needed collaboration between local authorities, primary care networks, social care, and community services to implement this vision. The goal is that existing vertical, siloed approaches to patient care, which do nothing to address those with complex multimorbidity, or complex social care needs, will be replaced with services that seamlessly join up around patient needs.

## Community Health and Wellbeing Workers

Of the recommended solutions that sites will be encouraged to adopt, the Community Health and Wellbeing Worker (CHWW) role epitomises this vision. Recruited from neighbourhoods, trained, and paid full or close to fulltime, CHWWs carry out monthly visits (or more frequently if needed) to each of the 120 or so households in their neighbourhoods, becoming the first-port-of-call for residents for advice, support, access to preventive services, and clinical care, joining up wider services and identifying problems early-on. Like seat-belts, CHWWs provide just-in-time support for when residents need it. CHWWs can help with whatever matters to the resident, whenever it matters to the resident. The role has been inspired by the Agentes Comunitarios de Saude system, which has been scaled throughout Brazil since the mid-1990s.^3,4^ Through over 20 years of research and advocacy,^5–7^ CHWWs were first piloted in Westminster in 2020 and, with the support of the National Association of Primary Care,^8^ have already spread into over 25 other localities around England. Currently, over 30 000 households in the most deprived areas in England are supported by CHWWs. Cornwall, Selby, Bristol, Torridge, Sutton, Plymouth, and Wandsworth are among the many places that have adopted the initiative. CHWWs are in a position to intimately understand the complex needs of all households and to direct services to them, and the results have been impressive. They have enabled rehousing of families mothballed for years on waiting lists; prevented attempted suicide in real-time; identified domestic violence; supported child carers into education; enabled the long-term unemployed back into employment; identified residents with undiagnosed hypertension, diabetes, depression, and dementia; increased the uptake of preventive services; reduced unscheduled primary care attendances and A&E attendances, as well as admissions to hospital.^9–11^

*“Like seat-belts, CHWWs provide just-in-time support for when residents need it. CHWWs can help with whatever matters to the resident, whenever it matters to the resident.”*

It is right, therefore, that the UK Government is recommending CHWWs for all localities. By putting the ‘neighbour’ into neighbourhood health care, and by ensuring a robust framework of professionalisation, training, and supervision, the far-reaching benefits that CHWWs bring means that, at scale, almost every statutory agency within the health and social care landscape stands to benefit from the CHWW role. The problem is that, because the funding landscape is still so fragmented and siloed, there is no funding solution that reflects the far-reaching, holistic approach that the CHWWs take. The pioneering sites that have embraced the CHWW role have experimented with a variety of funding solutions. Westminster drew first on public health funds to pump-prime the pilot, and then Primary Care Networks drew down the ARRS resources for the role. Cornwall’s ICB leveraged their recurrent Health Inequalities funds to support the role. In Plymouth, the British Red Cross is funding the role. Bridgewater used Community Transformation funding; Calderdale pivoted NHSE Community Mental Health Transformation funding through the voluntary sector. Each approach has advantages and disadvantages but what they all have in common is that they aren’t sustainable. The CHWW role requires a NATO-style approach to funding, where a very small proportion of funds can be top-sliced from different sectors, for example, housing, adult social care, acute trusts, and primary care, given they all stand to benefit directly or indirectly from a scaled CHWW approach. We estimate^12^ that just a 0.1% contribution from Education, Health, Housing, and the Public Order and Safety budgets^13^ would provide sufficient resources to fund CHWWs visiting all the households in the 20% most deprived areas of England each year. Alternatively, given their focus on prevention, just 10% of the Public Health budget could achieve this. Equally, the £2.2bn budget for health inequalities could fund the programme fully for 5 years in the 20% most deprived areas.

## Moving to a sustainable funding solution

National funding is being developed that will encourage ICBs to adopt neighbourhood initiatives such as CHWWs, but it is unlikely to become available for at least 12 months. That means around 15 or more active sites supporting approximately 100 CHWWs and some 20 000 residents in the most deprived areas will lose their programmes before any new funding mechanisms become available that can bridge the gap. Once the trust and relationships CHWWs have built are lost, they cannot simply be re-established later. For a section of our population that struggle to trust in services and are now engaging actively in managing their own health and wellbeing, if the programme goes and they lose their CHWWs, that hard-won fragile trust will be lost, meaning not just loss of the benefits gained to the individual but also reversal of the financial gains made by the system. When Jaguar Land Rover struggled to recover from a cyber-attack, the government underwrote a £1.5bn loan guarantee to safeguard around 30k jobs that were at risk. If the government is serious about delivering integrated neighbourhood health care they should apply the same thinking until they have developed the appropriate funding solutions to deliver on the ambition.
